# Neuronal Degeneration in Mice Induced by an Epidemic Strain of Saint Louis Encephalitis Virus Isolated in Argentina

**DOI:** 10.3389/fmicb.2018.01181

**Published:** 2018-06-07

**Authors:** María E. Rivarola, Soledad de Olmos, Guillermo Albrieu-Llinás, Laura B. Tauro, Melisa Gorosito-Serrán, Brenda S. Konigheim, Marta S. Contigiani, Adriana Gruppi

**Affiliations:** ^1^Laboratorio de Arbovirus, Instituto de Virología “Dr. J. M. Vanella”, Facultad de Ciencias Médicas, Universidad Nacional de Córdoba, Córdoba, Argentina; ^2^Laboratorio de Neuroanatomía e Histología Experimental, Instituto de Investigación Médica Mercedes y Martín Ferreyra – INIMEC-CONICET-UNC, Córdoba, Argentina; ^3^Instituto Nacional de Medicina Tropical, Ministerio de Salud, Puerto Iguazú, Argentina; ^4^Inmunología, Departamento de Bioquímica Clínica, Facultad de Ciencias Químicas, Universidad Nacional de Córdoba, Córdoba, Argentina

**Keywords:** Saint Louis encephalitis virus, neurotropic arbovirus, encephalitis, neurodegeneration, apoptosis

## Abstract

Saint Louis encephalitis virus (SLEV) is a neglected flavivirus that causes severe neurological disorders. The epidemic strain of SLEV, CbaAr-4005, isolated during an outbreak in Córdoba city (Argentina), causes meningitis and encephalitis associated with neurological symptoms in a murine experimental model. Here, we identified the affected brain areas and the damage triggered by this neurotropic arbovirus. We performed a detailed analysis of brain neurodegeneration associated with CbaAr-4005 SLEV infection in mice. The motor cortex, corpus striatum and cerebellum were the most affected structures. Neurodegeneration was also found in the olfactory bulb, thalamus, hypothalamus, hippocampus, and hindbrain. SLEV infection triggered brain cell apoptosis as well as somatodendritic and terminal degeneration. In addition, we observed massive excitotoxic-like degeneration in many cortical structures. Apoptosis was also detected in the neuroblastoma cell line N2a cultured with SLEV. The results evidenced that SLEV CbaAr-4005 infection induced severe degenerative alterations within the central nervous system of infected mice, providing new information about the targets of this flavivirus infection.

## Introduction

Saint Louis encephalitis virus (SLEV) is a neglected flavivirus that can cause severe neurological disease that may lead to death or sequelae. The pathogenesis of this virus is poorly understood, which hinders the development of a specific treatment or vaccine. Less than 1% of SLEV infections are evident and, because symptoms of infection are often mild or non-specific, diagnosis can be difficult. In acute SLEV infections, patients develop symptoms ranging from abrupt fever to more serious neurological complications, including stiff neck, confusion, disorientation, dizziness, tremors, unsteadiness, and post-infectious sequelae. The severity of clinical illness and the potential morbidity and mortality caused by SLEV increases with age, and people over 60 years old show higher frequency of encephalitis (data provided by the Centers for Disease Control and Prevention, United States).

Saint Louis encephalitis virus is widely distributed throughout the Americas. In past years, Argentina and Brazil have reported an increase in the number of cases of encephalitis associated with this virus (Spinsanti et al., 2003, 2008; Rocco et al., 2005; Mondini et al., 2007; Seijo et al., 2011), and the virus was detected in mosquito pools in California after an 11-year absence, concomitant with an Arizona outbreak (White et al., 2016). In Argentina, outbreaks were reported in the Provinces of Crdoba (2005) (Spinsanti et al., 2008), Entre Ros (2006), Buenos Aires (2010), and San Juan (2011) (Seijo et al., 2011). In 2005, 47 laboratory-confirmed clinical cases of SLEV infection, including 9 fatal cases, were reported in Córdoba, central Argentina (Spinsanti et al., 2008). In this scenario, the epidemic strain CbaAr-4005 (genotype III) was isolated from *Culex quinquefasciatus* mosquitoes collected around the house of a SLEV-infected patient (Díaz et al., 2006).

Most likely due to the few reports of SLEV infection during the decades preceding the reemergence, little is known about the neurological alterations induced by this virus. To address this fundamental gap in knowledge, we previously demonstrated that CbaAr-4005 strain of SLEV was more virulent (infective and lethal) than another strain isolated from a non-epidemic scenario (Rivarola et al., 2014). The CbaAr-4005 strain of SLEV was capable of generating high viremia levels, invading peripheral organs such as spleen and lungs, causing severe histopathological damage. Replication of this virus was also detected in the Central Nervous System (CNS), simultaneously with the appearance of disease symptoms such as excitability, tremor, and laterality in gait (Rivarola et al., 2017). However, we did not identify the CNS damage induced by SLEV inoculation.

In the present study, we make a detailed exploration of the neurodegeneration observed in mice infected with SLEV, reporting the type of cell death induced by virus inoculation and the different brain areas affected during the infection. The data may help understand the clinical consequences as well as the possible neurological sequelae of SLEV CbaAr-4005 infection.

## Materials and Methods

### SLEV Strain and Mice

The epidemic strain CbaAr-4005 (genotype III) was isolated from *Culex quinquefasciatus* mosquitoes collected from around the house of a Saint Louis Encephalitis -confirmed patient during a human encephalitis outbreak in 2005 in Córdoba (capital city of Córdoba province) (Díaz et al., 2006). The SLEV stocks used in this study were obtained after five rounds of propagation of the original isolates through suckling mice by means of brain inoculation. Seed stocks for our experiments were prepared by suspending 10% W/V Swiss albino mouse brain in Eagle’s minimum essential medium (MEM) supplemented with 10% fetal bovine serum (FBS) and 1% gentamicin. Virus stock titers were determined by standard Vero cell plaque assay and expressed as plaque forming units (Contigiani and Sabattini, 1987). Rockefeller Swiss albino mice, strain W1 (R-W1), of 21 days of age, were used; all experiments were performed in animal biosafety level 2 facilities. Inbred mice were housed and bred in the animal facility of the Arbovirus laboratory of the Virology Institute “Dr. J.M. Vanella” (Universidad Nacional de Córdoba).

### Viral Infection, Analysis of Morbidity, and Survival Curve

R-W1 mice (*n* = 5) were inoculated with 100 μL of viral suspensions containing 3000–5000 PFU/0.1mL of CbaAr-4005 strain. Infections were performed by subcutaneous injections to mimic mosquito bites. The control group consisted of mice (*n* = 5) inoculated with 100 μL of phosphate buffer saline (PBS). Mice were observed daily during 13 days post-infection (DPI) to record the number of dead individuals. As a measure of morbidity, signs of neurological illness (hindlimb paralysis, tremors, and laterality in gait) were also monitored daily during the same period. Both mortality and morbidity were expressed as percentages of the total analyzed mice. Blood samples were collected and sera (1:5 in MEM) were tested for anti-SLEV neutralizing antibodies (NTAb) using the plaque-reduction neutralization test (Contigiani and Sabattini, 1987).

### Study of Neurodegeneration: Infection, Perfusion, and Histological Procedure

Mice injected with CbaAr-4005 strain of SLEV (*n* = 10) or with PBS (control group, *n* = 5) were observed for symptoms during the infection and they were sacrificed at 8 DPI (when mice did not present signs of illness) or at 11 DPI (when mice were sick). Before euthanasia, blood samples were collected, and sera (1:5 in MEM) were tested for the detection of NTAb (Contigiani and Sabattini, 1987).

Mice were intraperitoneally anesthetized with chloral hydrate (0.3g/Kg) and transcardially perfused, rinsed with glucose (0.4%), sucrose (0.8%), and sodium chloride (0.8%), and fixed with 4% paraformaldehyde (PFA) in 0.2 M borate buffer (pH 7.4). Brains were immersed in 30% sucrose, and frontal and sagittal sections were cut at a thickness of 40 μm using a Cryostat (Leica Biosystems, Wetzlar, Germany). Four series of sister sections were stored either in 4% PFA for the amino-cupric-silver (A-Cu-Ag) technique (de Olmos et al., 1981), or in PBS 0.01M for the Fluoro-Jade B (FJB) staining (Chemicon, United States, Schmued and Hopkins, 2000). For Nissl and TUNEL techniques, brain sections (25 μm thick) were cut and directly mounted on gelatine-coated slides.

The A-Cu-Ag technique is highly sensitive for staining degenerating perikarya, dendrites, stem axons and their terminal arborizations in brain. The procedure was carried out following the protocol previously described (de Olmos et al., 1981). Briefly, sections were rinsed in double-distilled water and incubated in a pre-impregnating solution of silver nitrate at 50°C. After cooling to room temperature, sections were rinsed with acetone and transferred to a concentrated silver diamine solution for 40 min. Sections were then immersed in a formaldehyde/citric acid solution for 25 min and then the reaction was stopped in 0.5% acetic acid. Bleaching was done in two steps to eliminate the non-specific deposits of silver on the tissue, first in 6% potassium ferricyanide, washed in double distilled water, then transferred to 0.06% potassium permanganate for 20 s. After washing sections again, stabilization was done in 2% sodium thiosulphate, washed, transferred to a fixer solution for 1 min, and then the sections were mounted and placed on a slide warmer (30°C) until they were fully dry. The dry slides were cleared by immersion in xylene for 10 min before coverslipping. Sections were counterstained with Nissl to corroborate brain structures.

The fluorescein derivative, FJB, stains neurons undergoing degeneration. The technique was performed as described previously (Schmued and Hopkins, 2000). Briefly, brain sections were mounted on slides and immersed in a 1% sodium hydroxide solution (80% ethanol) for 5 min. Slides were then placed in 70% ethanol for 2 min and then rinsed in distilled water for 2 additional min. Subsequently, slides were transferred to a solution of 0.06% potassium permanganate for 10 min and then rinsed in distilled water for 2 min. The staining solution was prepared from a 0.01% stock solution of FJB that was prepared according to the manufacturer’s instructions. The stock solution was diluted by adding 0.1% acetic acid, resulting in a final dye concentration of 0.0004%. The working stain solution was prepared within 10 min of use and was not reused. Slides were stained for 20 min and then rinsed in distilled water (3 min × 1 min). The slides were then placed on a slide warmer (50°C) until they were fully dry. The dry slides were cleared by immersion in xylene for 2 min before coverslipping.

When mapping the neuropathological changes induced by the infection in the brain, we used the nomenclature proposed by Franklin and Paxinos (2007). For classifying neuronal damage, we used the criteria described in previous reports (de Olmos et al., 1981; Bueno et al., 2003). The neurons marked by coarse granular deposits on soma and surrounded by granular terminal-like deposits were referred to as “apoptotic-like degeneration.” Neurons with evident somatodendritic arghyrophilia showing a dark, smoothly contoured profile of soma and processes were considered as “somatodendritic degeneration.” Finally, axon terminal degeneration independent of the parent cell body death was termed “terminal degeneration.”

To evaluate neuronal degeneration, we quantified A-Cu-Ag stained sections of the olfactory bulb, cortical structures, caudate putamen and cerebellum of infected mice sacrificed at days 8 and 11 PI. Sections of uninfected mice were evaluated as controls. Images were obtained with a 40× lens in an optic microscope (OlympusCX40) equipped with a Zeiss AxioCam ERc5s video camera. Image analysis was performed using the ImageJ-FIJI software (Schindelin et al., 2012). A generalized linear model (GLM) using Poisson distribution was fitted to assess the contribution of the different groups (control, 8DPI, 11DPI) to the variations in the percentage of degenerated areas. This analysis was performed using the *MASS* package (Venables and Ripley, 1999) implemented in the R software (R Development Core Team, 2008).

### *In Situ* Apoptosis Detection

Sagittal sections of brains (25 μm) from infected (*n* = 5) and uninfected (*n* = 5) mice were obtained in a Cryostat (Leica Biosystems, Wetzlar, Germany) and directly mounted on gelatine-coated slides for apoptosis evaluation by the TUNEL (terminal deoxynucleotidyl transferase-mediated dUTP-biotin nick end labeling) assay, using a commercial kit (Roche Diagnostics, Mannheim, Germany) and by immunohistochemistry using an anti-cleaved caspase-3 antibody. For TUNEL assay, sections were hydrated in PBS for 30 min and permeabilized for 2 min with 0.1% Triton in 0.1% sodium citrate. After three washes with PBS, 20 μL of TUNEL mix containing 50 μL of terminal deoxynucleotidyl transferase (TdT) enzyme in 450 μL of dye solution was added. A negative control with 20 μL dye solution was used. Subsequently, sections were incubated in the dark in a humid chamber at 37°C, washed three times in PBS, and mounted with Fluor-Save (Sigma-Aldrich, St. Louis, MO, United States). For immunohistochemistry, sections were first incubated for 1 h at room temperature in a solution of 3% hydrogen peroxide and 10% methanol in PBS 0.01 M to quench endogenous peroxidase. Then the sections were washed in 0.01 M PBS and incubated in a blocking solution of 5% normal horse serum (NHS) for 1 h. After blocking, sections were incubated for 48 h at 4°C with a polyclonal anti-cleaved caspase-3 antibody (Cleaved-Asp175, Cell Signaling Technology, Danvers, MA, United States) diluted 1:400 in 0.01 M PBS containing 1% NHS and 0.03% Triton X100. Then, sections were washed in 0.01M PBS and incubated for 2 h in biotinylated secondary antibody (Vector Laboratories, Burlingame, CA, United States), washed in 0.01 M PBS, followed by an avidin-biotin-peroxidase complex (Vectastain ABC kit, Vector Laboratories) for 1 h at room temperature. Finally, sections were incubated for 5 min with a solution containing 0.05% 3-3′-diamino-benzidine tetra hydrochloride and 0.01% hydrogen peroxidase. Sections were mounted onto gelatine-coated slides, dehydrated and coverslipped.

### *In Vitro* Apoptosis Detection

The TUNEL assay was also performed in Neuro-2a (N2a) cells infected with SLEV CbaAr-4005 strain. For this, the N2a cells were grown on coverslips on 24-well plates containing supplemented MEM. Cells were counted by using an automated cell counter (Invitrogen, CA, United States) and infected with the 10^-4^ dilution of CbaAr-4005 SLEV (MOI = 1, selected according to cell viability determined by MTT) and incubated at 37°C for 1 h. Virus was removed and monolayers were washed with PBS. Supplemented MEM was added to each well and cells were incubated at 37°C. At 96 h post infection (HPI), 20 μL of TUNEL mix was added and the reaction was performed as described above. Micrographs were taken using an inverted fluorescence microscope (Olympus IX81, Imaging Software: Cell M) with rhodamine filter (653 nm), and positive cells (red) were counted in five fields. The number of apoptotic cells in infected and uninfected (control) cells was evaluated by generalized linear modeling (GLM) with negative binomial distribution and a log offset was applied to account for differences in the total number of cells. This analysis was performed using the *MASS* package (Venables and Ripley, 1999) in the R software (R Development Core Team, 2008).

### Immunofluorescence

Brains from SLEV-infected and control mice were taken at days 8 and 11 PI. They were cut into 25 μm thick sections and processed for viral detection by immunofluorescence. Briefly, slides were hydrated in PBS 0.1 M for 30 min and permeabilized with PBS-Triton (PBST) 0.25%. Subsequently, sections were washed three times with PBST-0.25% and PBST-0.1%, and incubated for 1.5 h in bovine serum albumin (BSA) 5% at 4°C. Brain sections were incubated overnight at 4°C with a MAB 6B6C-1antibody (1:20; Millipore, Massachusetts, United States) to detect the E protein of SLEV, washed three times with PBST-0,1%, and incubated with Goat Anti-Mouse IgG (1:600, Alexa Fluor^®^ 488). Finally, sections were washed with PBST-0.1%, counterstained with Hoechst, and mounted with Fluor-Save (Sigma-Aldrich, St. Louis, MO, United States). Micrographs were taken on a ZEISS Axioplan microscope coupled to a digital camera Leica DC 200.

### Ethics

All procedures were carried out following the institutional safety procedures (Area de Bioseguridad, Facultad de Ciencias Médicas, Universidad Nacional de Córdoba) and the guidelines of the Institutional Committee for the Care and Use of Laboratory Animals from the Facultad de Ciencias Médicas, Universidad Nacional de Córdoba, in accordance with the NIH-USA (2011) Guidelines for the Care and Use of Laboratory Animals and the EC Directive 86/609. The experimental procedures performed in our study were approved by the Institutional Committee (Identification number: 02/13-674/09).

## Results

**FIGURE 1:**
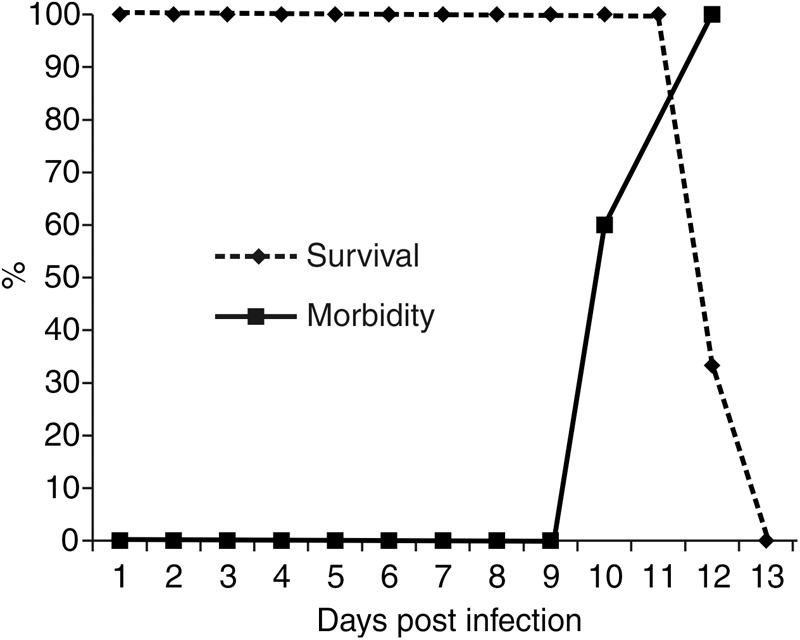
Survival and morbidity curves from R-W1 mice inoculated with SLEV CbaAr-4005 strain. A group of five mice were inoculated with 3000–5000 PFU/mL of SLEV by dorsal injection and followed until death. The survival curves were constructed using data from three independent experiments. This figure is representative of three independent experiments.

We previously reported that infection with SLEV CbaAr-4005 in R-W1 mice triggers behavioral symptoms, such as hindlimb paralysis, tremors and laterality in gait (Rivarola et al., 2017). In the present study, we observed that these symptoms began on day 10 PI, and by 13 DPI all animals died (**Figure 1**). Neither behavioral changes nor mortality were observed in the control group. The infection of inoculated mice was confirmed, in sera, by means of the plaque reduction neutralization test against SLEV (data not shown).

**FIGURE 2:**
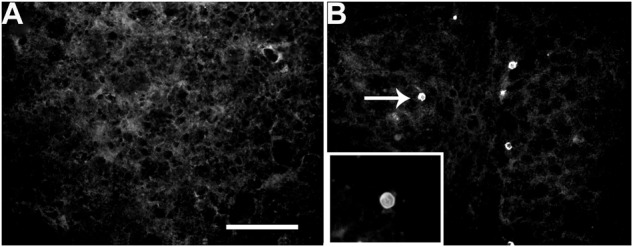
Detection of SLEV Cba-Ar-4005 E protein in the hippocampus of uninfected and infected mice, at day 11 PI. No specific immunostaining was observed in the uninfected mice **(A)**. The white arrow indicates an infected cell, where the viral E protein is marked with anti MAB8744, in the hippocampus of an infected mouse **(B)**. Bar = 50 μm. This figure is representative of three independent observations.

Taking into account that SLEV-infected mice present behavioral symptoms, we first evaluated the presence of the virus in different brain areas. Compared to uninfected mice (**Figure 2A**), by immunofluorescence we observed that RW-1 mice inoculated with CbaAr-4005 exhibited specific staining for SLEV E-protein in the hippocampus (**Figure 2B**), illustrating the presence of SLEV-infected cells. Although the frequency of immuno-positive cells was low, similar patterns were observed in different brain areas such as the olfactory bulb, cortical structures, hippocampus, caudate putamen, thalamus, hypothalamus, corpus callosum, cerebellum and brain stem (data not shown). The results confirmed the presence of the virus in brain.

We examined whether the infection was able to trigger neurodegeneration by evaluating the presence of neurological damage in different areas of the brain from uninfected and infected mice, either before the onset of behavioral symptoms (8 DPI) or the day before death (11 DPI). Results showing the histopathological analysis by A-Cu-Ag staining of the brains are summarized in **Table 1**. Different morphological expressions of degeneration were identified, such as apoptotic-like, somatodendritic and terminal degeneration in brain structures of mice sacrificed at days 8 and 11 PI.

**Table 1:** Type and degree of neuronal degeneration induced by SLEV CbaAr-4005 in brains of RW1 mice.

Brain region	Days post infection (DPI)	Apoptotic-like degeneration	Somatodendritic degeneration	Terminal degeneration
Olfactory bulb	8	+++	++	–
	11	+	+	–
Cortical structures	8	+	–	–
	11	+++	+++	–
Hippocampus	8	+	–	–
	11	+	–	+
Caudate putamen	8	+	–	–
	11	+++	+	++
Thalamus	8	+	–	–
	11	+++	+	–
Hypothalamus	8	+	–	–
	11	++	–	++
Corpus callosum	8	+	+	–
	11	++	–	++
Cerebellum	8	+	–	–
	11	+++	+++	+++
Brain stem	8	+	–	–
	11	++	+	–

*+++ severe damage, when the entire area of the brain showed degeneration. ++ moderate damage, when only a part of the area was affected. + scattered lesions, with reduced degeneration. - no damage at all.*

The most prominent degeneration was present at day 11 PI. Although all of the controls did not present degenerative silver staining, fragments of axons were occasionally observed in all analyzed areas. This phenomenon has been previously described in brain from rats as “incomplete staining of normal axons” (de Olmos et al., 1981).

**FIGURE 3:**
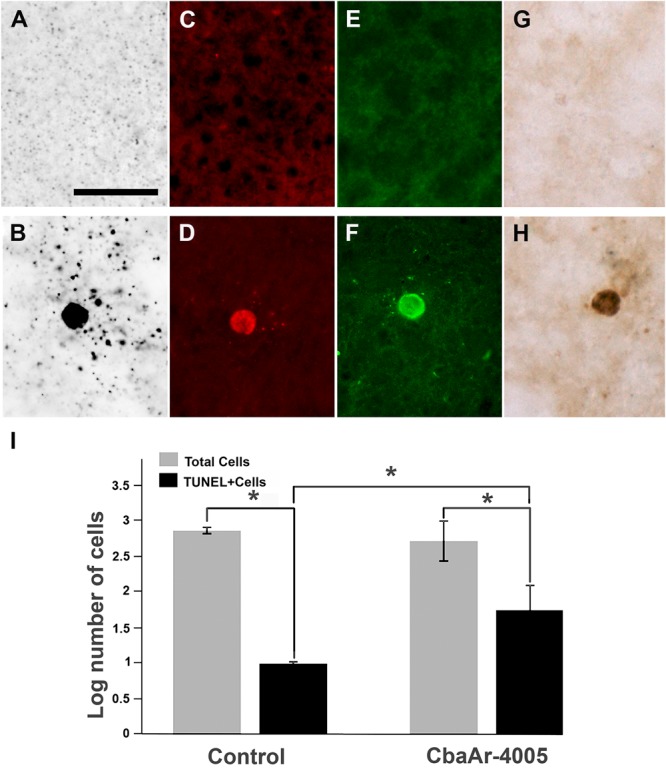
Apoptotic patterns in mouse brain and in N2a cell cultures induced by SLEV infection. Brain sections stained with A-Cu-Ag **(A,B)**, TUNEL **(C,D)**, Fluoro-Jade B **(E,F)** and anti-cleaved caspase-3 antibody **(G,H)** in uninfected **(A,C,E,G)** and SLEV strain CbaAr-4005 infected mice **(B,D,F,H)**. Bar = 10 μm. This figure is representative of three independent experiments. N2a cell cultures **(I)** were infected with SLEV (MOI = 1) and tested by TUNEL assay. (^∗^) Indicates a statistically significant variation in the number of apoptotic cells (*P* < 0.005). Total cells (gray bars) and apoptotic TUNEL+cells (black bars) are depicted.

To confirm the presence of apoptosis, the brains of uninfected (**Figures 3A,C,E,G**) were processed in parallel with the brains of SLEV-infected mice. The pattern of apoptotic-like cells in brain, revealed by the A-Cu-Ag, is shown in **Figure 3B**. We observed coarse granular deposits (black staining) in soma, surrounded by granular terminal-like deposits. This pattern was confirmed by TUNEL (**Figure 3D**), FJB (**Figure 3F**), and by immunostaining using anti-cleaved caspase-3 antibody (**Figure 3H**). The brains of uninfected mice showing the background signal of each staining is shown in **Figures 3A,C,E,G**. These results indicate that SLEV infection triggers apoptosis in cells from the brain.

To evaluate whether the virus directly induces apoptosis in neurons, N2a cells were infected with SLEV CbaAr-4005 (MOI = 1), which induced a 50% of cellular viability after 96 HPI according to a MTT test (not shown). By TUNEL, we confirmed that the number of apoptotic cells was significantly 5.6-fold higher in the cells incubated with the virus (*p* < 0.005), compared to cells cultured with medium alone (**Figure 3I**).

**FIGURE 4:**
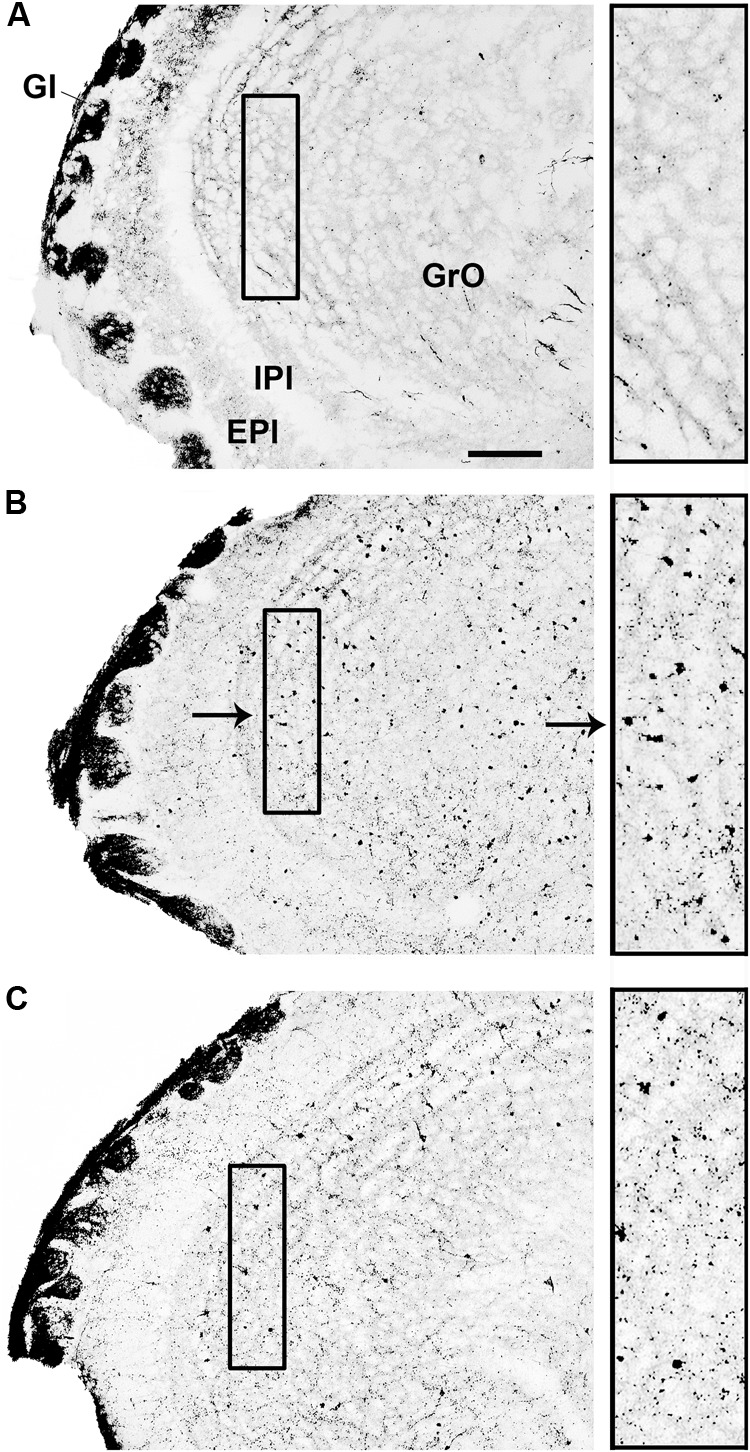
Infection with SLEV CbaAr-4005 induced numerous apoptotic-like A-Cu-Ag staining of cell bodies in the granule cell layer of the olfactory bulb (GrO). Compared to control mice **(A)**, mice sacrificed at day 8 PI showed a clear increase in apoptotic-like cells in GrO **(B)**. The increase in the number of apoptotic-like cells was less striking in mice sacrificed at day 11 PI than in those sacrificed at day 8 PI **(C)**. Insets show augmented details of the areas where SLEV induced apoptotic-like degeneration in GrO. Sagittal sections, lat.1.13; the black arrow in **(B)** indicates an apoptotic-like A-Cu-Ag stained cell. Bar = 100 μm. This figure is representative of three independent experiments. GI, glomerular layer of the olfactory bulb. IPI, internal plexiform layer of the olfactory bulb. EPI, external plexiform layer of the olfactory bulb.

The analysis of the different brain areas showed that infection with SLEV CbaAr-4005 induced numerous apoptotic-like cell bodies in the granule cell layer of the olfactory bulb (GrO), but not in its external plexiform layer (EPl) (**Figure 4**). Surprisingly, compared to controls (**Figure 4A**), the increase of apoptotic-like neurons in the GrO was more dramatic in the brain of mice with 8 DPI (**Figure 4B**) than in those sacrificed at day 11 PI (**Figure 4C**), indicating that apoptosis occurs before the onset of symptoms. Occasional somatodendritic degeneration in the periglomerular and granular layers of the main olfactory bulb was detected (data not shown). In 8 DPI mice, somatodendritic degeneration was also detected in the olfactory tubercle, dorsal endopiriform claustrum and piriform cortex (data not shown). At 11 DPI mice, layer I of the lateral olfactory tract, lateral anterior olfactory area, olfactory tubercle, Pir, and the different subdivisions of the entorhinal cortex presented terminal neurodegeneration (data not shown).

**FIGURE 5:**
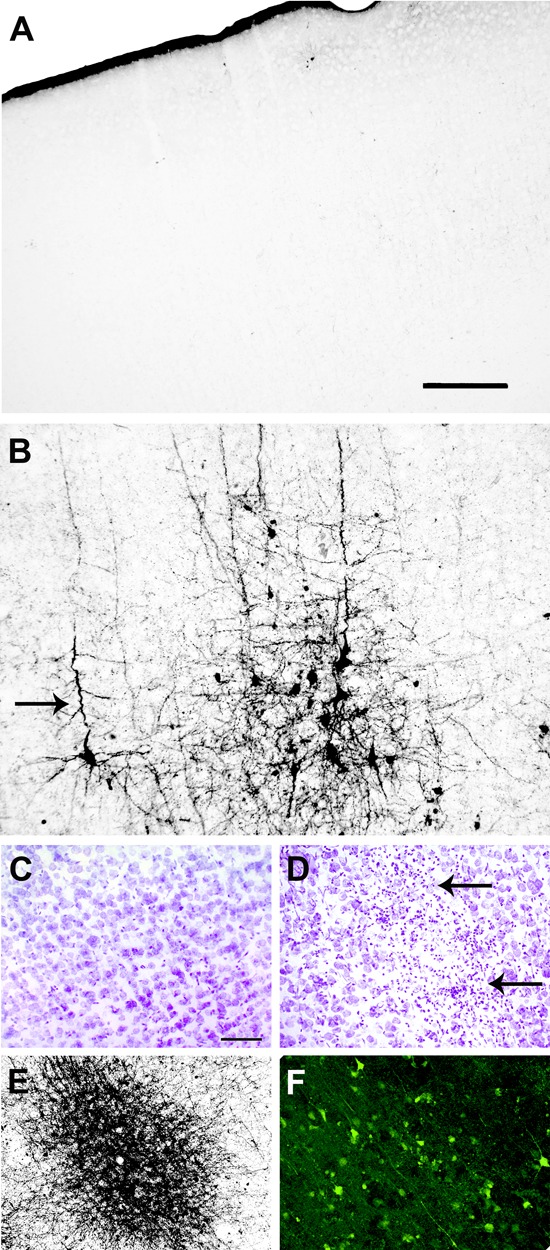
Infection with SLEV CbaAr-4005 induced exitotoxic-like degeneration in cortical structures of mice sacrificed at day 11 PI. Compared to the brain of uninfected mice **(A)**, the motor cortex from the brain of infected mice presented various degenerating patterns, with neurons with golgi-like appearance with smooth contours, homogeneous impregnation of the cell bodies and corkscrew dendritic arbors (arrow), surrounded by apoptotic-like neurons **(B)**; Bar = 50 μm. Consecutive sections of brains of control **(C)** and infected **(D)** mice, stained with Nissl, A-Cu-Ag **(E)** and Fluoro-Jade B **(F)**, also evidenced degeneration with cell loss and gliosis (small arrows). Sagittal sections Lat 2.10, Bar = 50 μm. This figure is representative of three independent experiments.

In different cortical structures, the overall degeneration induced by viral CbaAr-4005 infection was practically undetectable in 8 DPI mice, except for sparsely scattered apoptotic-like neurons (not shown). However, compared to the brain of uninfected mice (**Figure 5A**), mice sacrificed at day 11 PI had many cortical structures with massive exitotoxic-like degeneration that could be easily observed (**Figure 5B**). The structure that presented most degeneration was the motor cortex, which revealed various degenerating patterns, neurons with golgi-like appearance with smooth contours, homogeneous impregnation of the cell bodies and corkscrew dendritic arbors, surrounded by apoptotic-like neurons (**Figure 5B**). Compared to controls (**Figure 5C**), consecutive sections of the brain of 11 DPI mice stained with Nissl presented gliosis (**Figure 5D**). A-Cu-Ag and FJB cells also evidenced degeneration with cell loss (**Figures 5E,F**, respectively). Other cortical areas such as the frontal association cortex and the prelimbic cortex presented a strong argyrophilic reaction and fibers were so dense that it was impossible to distinguish degenerating cell bodies and processes.

Comparatively, hippocampal structures did not present such dramatic degeneration patterns as the cortex. However, scattered apoptotic-like degeneration was observed in both 8 and 11 DPI mice (**Table 1**), where most apoptotic-like neurons were found in field CA2 of the hippocampus, the molecular layer of the dentate gyrus, the granule cell layer of the dentate gyrus, and the dorsal subiculum (not shown). Terminal degeneration was detected in the CA1 stratum lacunosum moleculare, stratum lucidum of CA3/CA4, and in the subicular complex. Additional terminal degeneration was observed in perirhinal and ectorhinal cortices (not shown).

**FIGURE 6:**
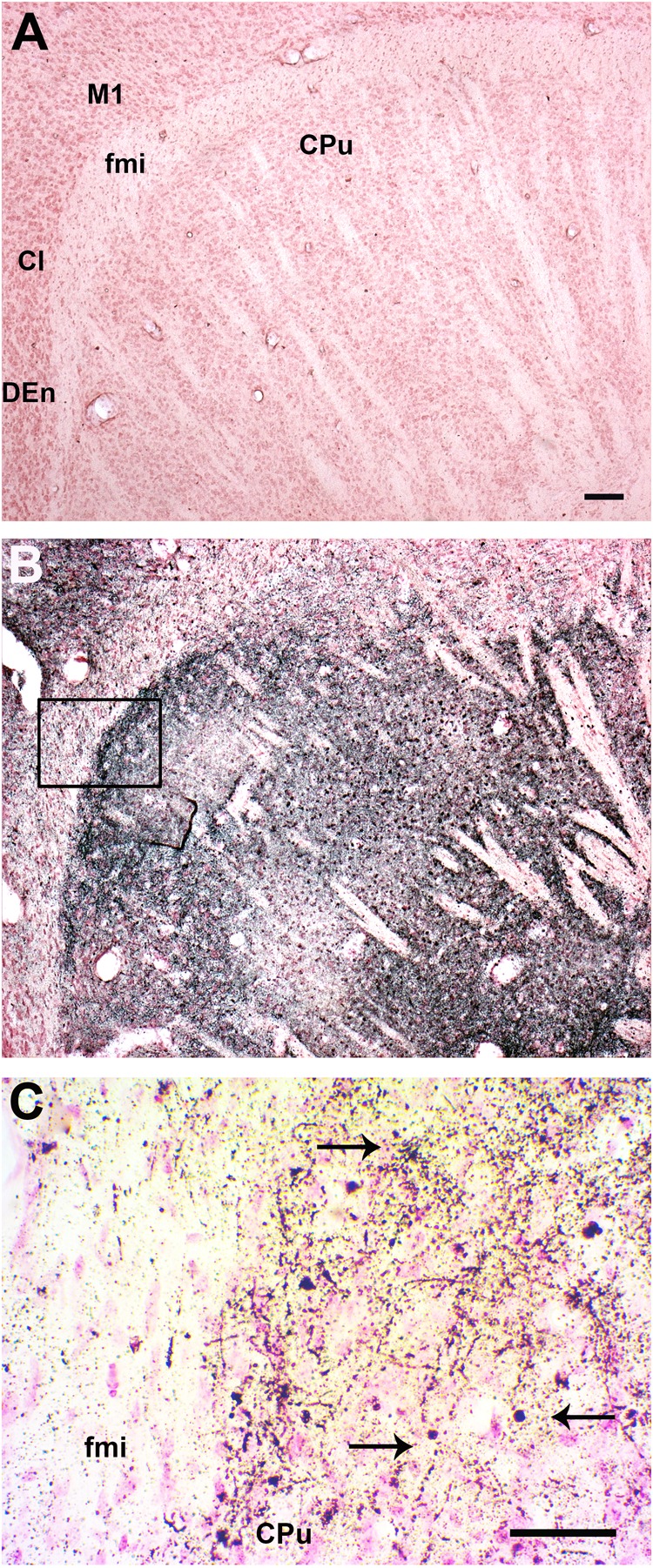
Infection with SLEV CbaAr-4005 induced degeneration in the Caudate Putamen. Compared to the brain of uninfected mice **(A)**, a remarkable increase in the density of A-Cu-Ag stained fibers was clearly observed in the striatum, along with clusters of apoptotic-like cells and even occasional somatodendritic degeneration in mice sacrificed at day 11 PI **(B)**. An augmented detail of the area affected by apoptotic-like degeneration (inset in **B**) is shown in **(C)**; black arrows indicate A-Cu-Ag stained cells. Sagittal sections Lat.3.4, Bar = 100 μm. This figure is representative of three independent experiments. M1, primary motor cortex. fmi, forceps minor of the corpus callosum. CPu, caudate putamen (striatum). CI, claustrum. DEn: dorsal endopiriform claustrum.

Mice sacrificed at day 8 PI presented only scattered apoptotic-like cells in the caudate putamen (CPu, striatum), accompanied by an overall increase in silver-stained fibers (**Table 1**). In comparison to uninfected mice (**Figure 6A**), at day 11 PI a remarkable increase in the density of clusters of A-Cu-Ag stained fibers was observed (**Figure 6B**). An augmented detail of the area affected by apoptotic-like degeneration (inset in B) is shown in **Figure 6C**.

In mesencephalic and diencephalic structures of the brain, such as the thalamus, an increased number of apoptotic-like cells were observed in 11 DPI mice and, to a lesser degree, in 8 DPI mice (**Table 1**). In the hypothalamus, not only apoptotic-like cells but also terminal degeneration was observed.

Terminal degeneration was also present in the corpus callosum, where a moderate amount of silver-stained fiber along with apoptotic cell death was observed in 11 DPI mice (**Table 1**). Other areas of the brain, such as layer I of the lateral olfactory tract, lateral anterior olfactory area, olfactory tubercle, Pir, and the different subdivisions of the entorhinal cortex, also presented terminal degeneration.

**FIGURE 7:**
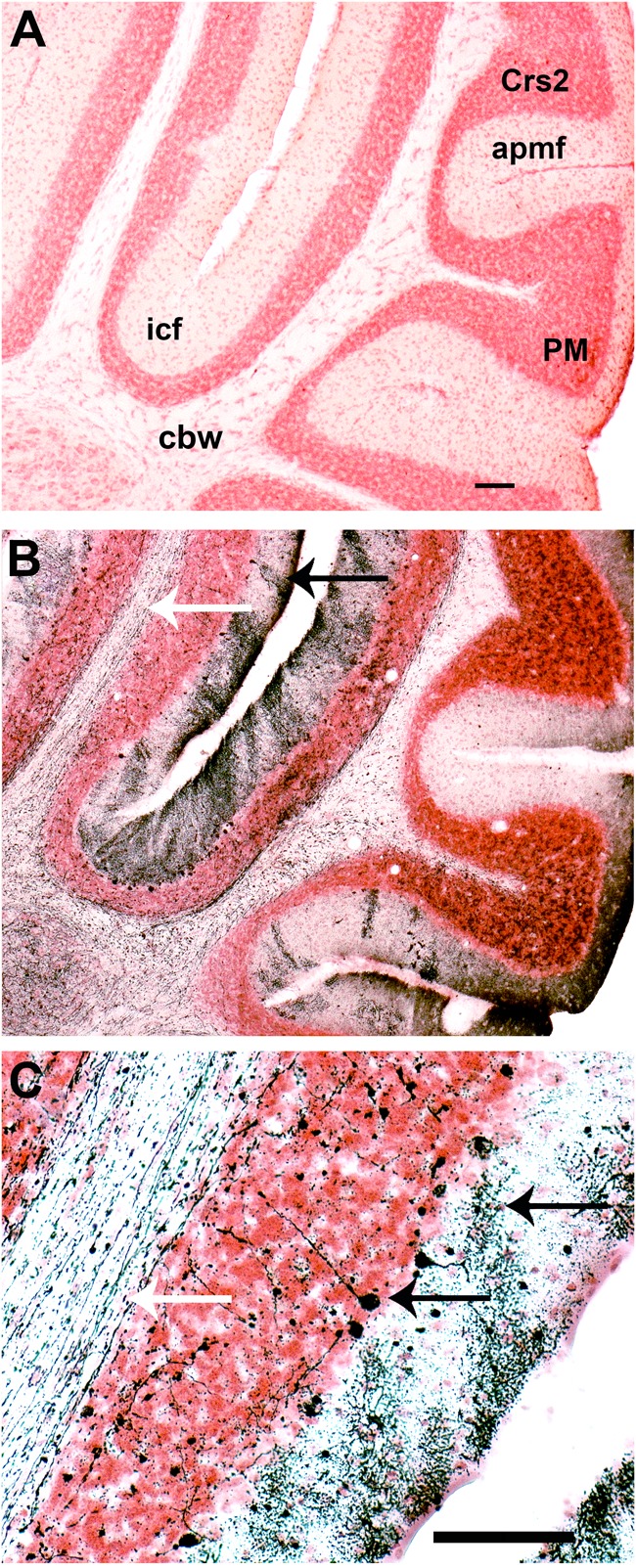
Infection with SLEV CbaAr-4005 induced neurodegeneration in mouse cerebellum. Compared to the brain of uninfected mice **(A)**, degeneration is seen in cerebellum fibers of mice sacrificed at day 11 PI (**B,C**; white arrows), and degenerated dendrites were identified (**B**: black arrow; **C**: upper black arrow). Purkinje cells underwent somatodendritic degeneration in the cerebellum (**C**: lower black arrow). This figure is representative of three independent experiments. Crs2, crus 2 of the ansiform lobule. apmf, ansoparamedian fissure. icf, intercrural fissure. PM, paramedian lobule. cbw, cerebellar white matter. Sagittal section Lat 2.62 Bar = 100 μm **(A,B)**; Bar = 50 μm **(C)**.

Finally, degeneration by apoptosis was also visible in the cerebellum and brain stem at both 8 and 11 DPI (data not shown). Compared to control (**Figure 7A**), there was a great amount of fiber tract degeneration in mice sacrificed at day 11 PI, with dense degeneration of dendrites in the molecular layer of the intercrural fissure (icf, **Figures 7B,C**, white arrow) and axons in the cerebellar white matter (cbw, **Figure 7B**, black arrow, **Figure 7C**, black upper arrow). Degenerated Purkinje cells were also identified in the superficial part of the granular layer in the cerebellum (**Figure 7C**, black lower arrow).

**FIGURE 8:**
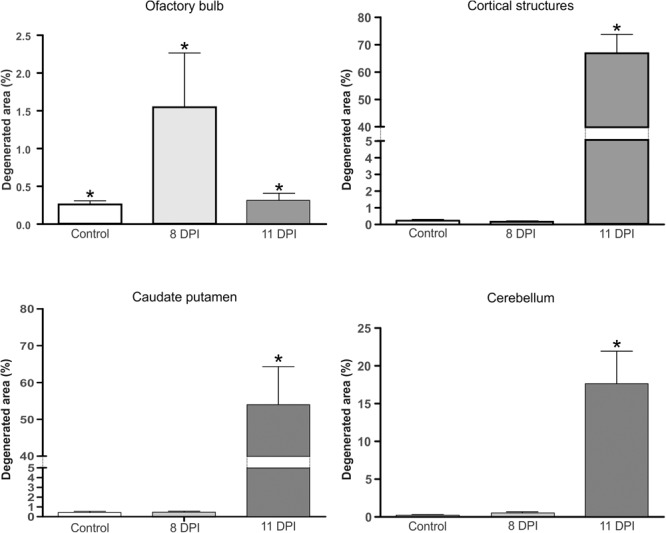
Percentage of the neurodegenerated area in brains of 8 (light gray bars) or 11 DPI (dark gray bars) mice, revealed by A-Cu-Ag staining, in olfactory bulb, cortical structures, caudate putamen, and cerebellum. Results are representative of three independent experiments, and uninfected mice were included as controls (white bars). (^∗^) Indicates a statistically significant association between the percentage of degenerated areas and the different mice models (control, 8 DPI, and 11 DPI) (*P* < 0.005).

**Figure 8** shows the statistical analysis of the degenerated sections of the brain areas showed in **Figures 4–7**.

**FIGURE 9:**
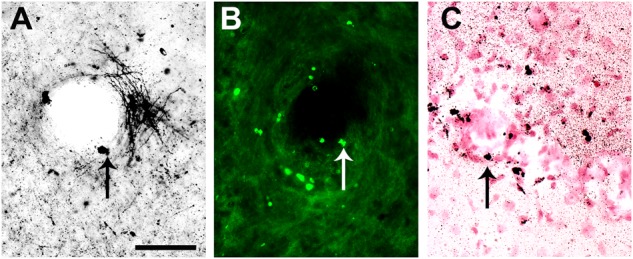
Capillaries in cortical structures bordered by apoptotic-like in a mouse infected with SLEV CbaAr-4005. Capillaries in cortical structures were densely bordered by apoptotic-like neurons, with cell bodies showing nuclear condensation with A-Cu-Ag **(A)** and Fluoro-Jade B **(B)** staining. Counterstaining A-Cu-Ag with Nissl confirmed presence of capillaries **(C)**. Bar = 50 μm. This figure is representative of three independent experiments.

It is interesting to point out that many capillaries in cortical structures of brain were densely bordered by apoptotic-like neurons, with cell bodies showing nuclear condensation with both A-Cu-Ag and FJB staining (**Figures 9A,B**); this phenomenon was more pronounced in medial and caudal sections. The capillaries were revealed by counterstaining A-Cu-Ag with Nissl (**Figure 9C**).

## Discussion

Saint Louis encephalitis outbreaks have been occurring in South America during the past decade and, recently, SLEV reemerged in California, concomitant with an Arizona outbreak (White et al., 2016). The California and Arizona SLEV isolates share >99% nucleotide identity with their closest published relative, the Argentinian strain CbaAr-4005, which was used as a neuropathogenic model in the present study.

To enhance our knowledge of SLEV neuropathogenesis, we focused the present study on detailed analyses of the brain damage induced by the epidemic strain CbaAr-4005 using a murine model. The first neuropathological signs began on day 10 PI but were markedly higher after day 11 PI, concordantly with the time of death (**Figure 1**) and the most prominent neurodegeneration observed. These behavioral alterations were characterized by hindlimb paralysis, laterality in gait and kinetic tremors. By using A-Cu-Ag, FJB and TUNNEL techniques and the detection of cleaved caspase-3, we determined that the neurodegeneration involved apoptotic, somatodendritic and terminal degeneration (**Table 1**). The motor cortex (**Figure 5**), corpus striatum (**Figure 6**) and cerebellum (**Figure 7**) were the most affected brain areas, suggesting that the symptomatology may be related to the damage described in these areas. To a lesser degree, we detected neurodegeneration in the olfactory bulb, thalamus, hypothalamus, hippocampus, and hindbrain. Interestingly, the olfactory bulbs of mice sacrificed at day 8 PI were more affected than those of mice harvested at day 11 PI (**Figures 4, 8**), which is in concordance with a protective mechanism of viral clearance within the olfactory bulb, as observed by other authors during neuroinvasive infections (Durrant et al., 2016). In this study, we did not address the mechanisms by which SLEV CbaAr-4005 invades the CNS; however, the early damage observed in the olfactory bulb could indicate invasion in this area during the first steps of infection of the CNS. Similarly, it has been shown that WNV and JEV are first detected within the olfactory bulb during neuroinvasive infection (Monath et al., 1983; Brown et al., 2007).

Although damage in the CNS after infection of mice with other flaviviruses has been previously described, these efforts were mainly focused on the effect of the infiltration of immune cells from the periphery into the CNS at different stages of the disease (Chiba et al., 1999; Shrestha et al., 2003; Hayasaka et al., 2009; Kimura et al., 2010; Donadieu et al., 2013; Lim et al., 2013). In our study, we specifically analyzed the different types and degree of neurodegeneration in numerous structures of the CNS. Particularly, we showed that SLEV infection induces apoptotic-like degeneration *in vivo*, and we also demonstrated a direct association between viral replication and apoptosis induction in N2a cells (**Figure 3**). In a previous study we also observed that the appearance of apoptosis in brain was associated with increased SLEV CbaAr-4005 titers (Rivarola et al., 2017), suggesting that neurons undergo cell death as a direct result of viral replication. However, we cannot rule out viral replication in brain triggering the production of pro-inflammatory cytokines and proapoptotic molecules that contribute to cell death, as previously shown in a mouse model after intracranial inoculation (Marquez et al., 2017). There is strong evidence that infection with flaviviruses activates apoptotic mechanisms in neurons, *in vivo* and/or *in vitro* (Hase et al., 1990; Marianneau et al., 1998; Weissenbock et al., 2000; Parquet et al., 2001; Raung et al., 2001; Prikhod’ko et al., 2002; Yang et al., 2002; Shrestha et al., 2003; Wang et al., 2003; Samuel et al., 2007), confirming that the induction of programmed cell death is a common feature after flavivirus infection. Although we detected apoptotic neurons, we cannot rule out the occurrence of other types of death mechanisms, like those described by authors who found necrotic neurons in the brain of mice infected with CHIKV and Western Equine Encephalitis virus (WEEV) (Powers and Logue, 2007; Logue et al., 2009).

Our study also showed localized apoptotic foci as well as viral particles in the periphery of blood vessels (**Figure 9**). Similarly, a previous histological analysis of the brain of mice infected with SLEV CbaAr-4005 showed inflammatory infiltrate in the meninges and sporadic inflammatory infiltrate around blood vessels (Rivarola et al., 2017). Our results may indicate that SLEV enters the CNS from the circulatory system to the mouse brain, but further studies are necessary to elucidate the mechanism by which SLEV crosses the blood–brain barrier.

In addition to apoptotic-like neurodegeneration, we observed massive excitotoxic-like degeneration in many cortical structures of mice sacrificed at day 11 PI (**Figure 5**), suggesting that the virus causes bystander damage through a glutamate-mediated excitotoxic mechanism. Chronic and acute viral infections contribute to the release of an excess of excitatory amino acids, such as glutamate, increasing the stimulation of specific receptors and consequently leading to lethal metabolic derangement and excitotoxic death of mature neurons (Nargi-Aizenman et al., 2004; Chen et al., 2012). For example, Chen et al. (2012) suggested a potential glutamate-mediated excitoneurotoxic mechanism after infection with JEV, which could be linked to neuronal injury and death of many cortical pyramidal neurons. Nargi-Aizenman et al. (2004) also showed that glutamate receptor stimulation is a major contributor to neuronal cell death caused by infection with Neuroadapted Sindbis virus (NSV), and that neuronal survival is increased by treatment with glutamate receptor antagonists. Our study also revealed an excitotoxic-like pattern in purkinje cells of the cerebellum (**Figure 7**) and the caudate putamen which could be related to many of the behavioral alterations observed at day 11 PI.

## Conclusion

The therapeutic options for SLEV and other flavivirus-induced CNS diseases are limited and no specific treatments of proven benefit are currently available. In the present study, we detailed the neuropathogenesis induced by SLEV in mice by screening different types of neuronal degeneration and these observations could be helpful to identify therapeutic targets to mitigate the neurological damage induced by this virus.

## Author Contributions

MR, AG, and MC conceived and designed the study. MR, SdO, GA-L, LT, MG-S, and BK performed the experiments. MR, SdO, GA-L, and AG analyzed the data and drafted the manuscript. All authors read and approved the final manuscript.

## Conflict of Interest Statement

The authors declare that the research was conducted in the absence of any commercial or financial relationships that could be construed as a potential conflict of interest.
